# Supplementary material for: Control of snakebite envenoming: A mathematical modeling study
Source: PLoS Negl Trop Dis. 2021 Aug 27;15(8):e0009711. doi: 10.1371/journal.pntd.0009711 (PMC8428672; doi:10.1371/journal.pntd.0009711)
Supplement: S1 File — (PDF) [file pntd.0009711.s001.pdf]

S1 File. Model Description. The population of unaware susceptibles is generated by constant recruitment rate given by Λ_H and diminishes by enlighten the individuals at a rate ϵ, snakebite envenomation at a rate λ, and natural death at a rate μ_H. Thus, the equation governing the dynamics of unaware susceptible sub-population is given by

$$\frac{dS_U}{dt} = \Lambda_H - (\epsilon + \lambda + \mu_H)\, S_U, \tag{1}$$

where,

$$\lambda(t) = \frac{\beta N_S}{N_H + N_S}. \tag{2}$$

In eq(2), β, is the effective snakebite envenomation rate. The population of aware susceptible individuals is increased when unaware individuals are educated at a rate ϵ and by those who recovered and assumed to take necessary measures in order to avoid another bite at the rates ϕ_1 and ϕ_2 respectively, in R_D and R_W compartments . This sub-population reduces due to envenoming at a reduced rate ($(1-\theta)\lambda$), in comparison with the unaware susceptible individuals, S_U, where $0 \le \theta \le 1$ measures the efficacy of public health awareness in reducing snakebite envenoming. If $\theta = 0$, the public health awareness has no effect on the behavior of individuals, while if $\theta = 1$ then the public health awareness campaign is 100% effective. The aware susceptible population further diminishes due to natural mortality at a rate μ_H. Thus, we have

$$\frac{dS_E}{dt} = \epsilon S_U + \phi_1 R_D + \phi_2 R_W - [(1-\theta)\,\lambda + \mu_H]\, S_E. \tag{3}$$

The population of SBE individuals is generated as a result of envenoming acquired by individuals in S_U and S_E compartments at a proportion given by $[(1-\theta)\, S_E + S_U)]\, \lambda$. The population is decreased due to treatment at a rate τ, snakebites envenoming induced mortality rate, δ_1 and natural mortality at the rate, μ_H. Thus,

$$\frac{dI}{dt} = [(1-\theta)\, S_E + S_U]\, \lambda - (\tau + \delta_1 + \mu_H)\, I. \tag{4}$$

The population of individuals receiving early treatment T_E, is generated by the proportion of envenomed individuals receiving early treatment at a rate τk and diminishes by early adverse reaction at a rate α_1, recovery without disability at a rate, γ_1 and natural mortality at the rate, μ_H. Thus, we have

$$\frac{T_E}{dt} = \tau k I - (\alpha_1 + \gamma_1 + \mu_H)\, T_E. \tag{5}$$

The population of individuals receiving late treatment is generated by proportion, $1-k$, of the envenomed individuals receiving late treatment at a rate, $\tau(1-k)$. This population reduces by early adverse reaction at a rate α_2, recovery with disability at a rate, σ_1, envenoming induced death at a rate, δ_2 and natural mortality at a rate μ_H. Hence, the equation governing the dynamics of T_L is given by,

$$\frac{T_L}{dt} = \tau(1-k)I - (\alpha_2 + \sigma_1 + \delta_2 + \mu_H)\, T_L. \tag{6}$$

The population of individuals suffering from early adverse reaction during early treatment, ($V_E(t)$), is generated by early adverse reaction at a rate α_1, and diminishes by recovery without disability at a rate, γ_2 and natural mortality at the rate μ_H. Thus, we obtain

$$\frac{V_E}{dt} = \alpha_1 T_E - (\gamma_2 + \mu_H)\, V_E. \tag{7}$$

The population of individuals suffering from early adverse reaction during late treatment, $(V_L(t))$, is generated by early adverse reaction at a rate α_2, and decreases by recovery with or without disability at a rate, σ_2, envenoming induced death at the rate, δ_2 and natural mortality at the rate μ_H. Thus, we have

$$\frac{V_L}{dt} = \alpha_2 T_L - (\sigma_2 + \delta_2 + \mu_H) V_L. \tag{8}$$

The population of individuals who recovered with disabilities, $(R_D(t))$ is generated by the proportions of individuals who recovered with disabilities in T_L and V_L compartments at the rates $\sigma_1\rho_1$ and $\sigma_2\rho_2$, respectively. It diminishes because of movement to S_E compartment at the rate ϕ_1 and natural mortality at the rate μ_H. Therefore, the equation governing the dynamics of individuals in R_D is given by

$$\frac{R_D}{dt} = \sigma_1\rho_1 T_L + \sigma_2\rho_2 V_L - (\phi_1 + \mu_H) R_D. \tag{9}$$

The population of individuals who recovered without disabilities, $(R_W(t))$, is generated by the proportions of individuals who recovered without disabilities in T_E, T_E, T_L and V_L compartments at the rates $\gamma_1, \gamma_2, \sigma_1(1-\rho_1)$ and $\sigma_2(1-\rho_2)$, respectively. It reduces as the result of transition to S_E compartment at the rate ϕ_2 and natural mortality at the rate μ_H. Thus, we have

$$\frac{R_W}{dt} = \gamma_1 T_E + \gamma_2 V_E + \sigma_1(1-\rho_1)T_L + \sigma_2(1-\rho_2)V_L - (\phi_2 + \mu_H)R_W. \tag{10}$$

The snake population increases at a logistic growth rate, $\Lambda_S N_S\left(1 - \frac{N_S}{K_S}\right)$, where, Λ_S and K_S are the recruitment rate of snake and population carrying capacity respectively. It reduces by natural mortality of the snake at a rate μ_S. Thus we have

$$\frac{dN_S}{dt} = \Lambda_S N_S\left(1 - \frac{N_S}{K_S}\right) - \mu_S N_S. \tag{11}$$

Let D be the cumulative number of deaths induced by snakebite. Thus, we have

$$\frac{dD}{dt} = \delta_1 I + (T_L + V_L)\delta_2. \tag{12}$$
