# Supplementary material for: Control of snakebite envenoming: A mathematical modeling study
Source: PLoS Negl Trop Dis. 2021 Aug 27;15(8):e0009711. doi: 10.1371/journal.pntd.0009711 (PMC8428672; doi:10.1371/journal.pntd.0009711)
Supplement: S2 File — (PDF) [file pntd.0009711.s002.pdf]

S2 File. Existence of an Optimal Control.

Consider the optimal control model

$$\begin{aligned}
\frac{dS_U}{dt} &= \Lambda_H - (\lambda + \epsilon u_1(t) + \mu_H)\, S_U, \\
\frac{dS_E}{dt} &= \epsilon u_1(t) S_U + \phi_1 R_D + \phi_2 R_W - ((1-\theta)\,\lambda + \mu_H)\, S_E, \\
\frac{dI}{dt} &= ((1-\theta)\, S_E + S_U)\,\lambda - (\tau u_2(t) + \delta_1 + \mu_H)\, I, \\
\frac{dT_E}{dt} &= \tau u_2(t) k I - (\alpha_1 + \gamma_1 + \mu_H)\, T_E, \\
\frac{dT_L}{dt} &= \tau u_2(t)\,(1-k)\, I - (\alpha_2 + \sigma_1 + (1-u_2(t))\delta_2 + \mu_H)\, T_L, \\
\frac{dV_E}{dt} &= \alpha_1 T_E - (\gamma_2 + \mu_H)\, V_E, \\
\frac{dV_L}{dt} &= \alpha_2 T_L - (\sigma_2 + (1-u_2(t))\delta_2 + \mu_H)\, V_L, \\
\frac{dR_D}{dt} &= \sigma_1 \rho_1 T_L + \sigma_2 \rho_2 V_L - (\phi_1 + \mu_H) R_D, \\
\frac{dR_W}{dt} &= \gamma_1 T_E + \gamma_2 V_E + \sigma_1 \Pi_3 T_L + \sigma_2 \Pi_4 V_L - (\phi_2 + \mu_H) R_W, \\
\frac{dN_S}{dt} &= \Lambda_S N_S \left(1 - \frac{N_S}{K_S}\right) - \mu_S N_S, \\
\frac{dD}{dt} &= \delta_1 I + (T_L + V_L)(1-u_2(t))\delta_2, \\
\lambda(t) &= \frac{\beta N_S}{N_H + N_S},
\end{aligned} \tag{13}$$

with the following objective functional

$$J(u_1, u_2) = \int_0^T \left(B_1 I(t) + B_2 D(t) + \frac{1}{2} \sum_{i=1}^{2} \left(C_i u_i^2 \right) \right) dt. \tag{14}$$

Theorem 0.1. *Consider the objective functional J given by eq (14) with the controls $(u_1, u_2) \in \Psi$ subject to the constraint state system in eq (13). There exists $(u_1^*, u_2^*) \in \Psi$ such that $J(u_1^*, u_2^*) = \min\{J(u_1, u_2), (u_1, u_2) \in \Psi\}$.*

Proof. The prove of this theorem is established by verifying the properties of Theorem 4.1 and its corresponding corollary by Fleming and Rishel in [1]. Also see the following articles [3, 4] for detail proof of the theorem. □

Optimalility System

Having established the existence of optimal controls pair, next step is to apply Pontryagin's maximum principle in [2]. This will allow for the derivation of the necessary conditions that must be satisfied by the pair of optimal controls and its corresponding state variables. The Lagrangian of the system which is the integrand of the objective functional is given by;

$$L = B_1 I + B_2 D + \frac{1}{2} \sum_{i=1}^{2} \left(C_i u_i^2 \right). \tag{15}$$

The Hamiltonian of the optimal control problem which comprises of the integrand of the objective functional and the inner product of the right hand side of the state

system in eq (13) and the adjoint variables $\lambda_i, (i = 1, 2, ..., 11)$ define as,

$$
\begin{aligned}
H =& B_1 I + B_2 D + \frac{1}{2}\sum_{i=1}^{2}\left(C_i u_i^2\right) + \lambda_1 \frac{dS_u}{dt} + \lambda_2 \frac{dS_e}{dt} + \lambda_3 \frac{dI}{dt} + \lambda_4 \frac{dT_E}{dt} + \lambda_5 \frac{dT_L}{dt} + \lambda_6 \frac{dV_E}{dt} \\
&+ \lambda_7 \frac{dV_L}{dt} + \lambda_8 \frac{dR_D}{dt} + \lambda_9 \frac{dR_W}{dt} + \lambda_{10} \frac{dN_S}{dt} + \lambda_{11} \frac{dD}{dt}, \\
=& B_I + B_2 D + \frac{1}{2}(C_1 u_1^2 + C_2 u_2^2) \\
&+ \lambda_1 \left[\Lambda_H - \left(\lambda + \epsilon u_1(t) + \mu_H\right) S_U\right] \\
&+ \lambda_2 \left[\epsilon u_1(t) S_U + \phi_1 R_D + \phi_2 R_W - \left(\left(1-\theta\right)\lambda + \mu_H\right) S_E\right] \\
&+ \lambda_3 \left[\left(\left(1-\theta\right) S_E + S_U\right)\right) \lambda - \left(\tau u_2(t) + \delta_1 + \mu_H\right) I\right] \\
&+ \lambda_4 \left[\left(\tau u_2(t)\right) kI - \left(\alpha_1 + \gamma_1 + \mu_H\right) T_E\right] \\
&+ \lambda_5 \left[\left(\tau u_2(t)\right)(1-k)I - \left(\alpha_2 + \sigma_1 + (1-u_2(t))\delta_2 + K_2\right) T_L\right] \\
&+ \lambda_6 \left[\alpha_1 T_E - \left(\gamma_2 + \mu_H\right) V_E\right] \\
&+ \lambda_7 \left[\alpha_2 T_L - \left(\sigma_2 + (1-u_2(t))\delta_2 + \mu_H\right) V_L\right] \\
&+ \lambda_8 \left[\sigma_1 \rho_1 T_L + \sigma_2 \rho_2 V_L - (\phi_1 + \mu_H) R_D\right] \\
&+ \lambda_9 \left[\gamma_1 T_E + \gamma_2 V_E + \sigma_1 (1-\rho_1) T_L + \sigma_2 (1-\rho_2) V_L - (\phi_2 + \mu_H) R_W\right] \\
&+ \lambda_{10} \left[\Lambda_S N_S \left(1 - \frac{N_S}{K_S}\right) - \mu_S N_S\right] \\
&+ \lambda_{11} \left[\delta_1 I + (T_L + V_L)(1-u_2(t))\delta_2\right].
\end{aligned} \tag{16}
$$

The adjoint system and the control characterization is presented in the following theorem. For computational convenience, let $f = \frac{1}{N_H + N_S}$.

Theorem 0.2. *Let $S_U^*, S_E^*, I^*, T_E^*, T_L^*, V_E^*, V_L^*, R_D^*, R_W^*$, N_S^* and D^* be the solutions of the corresponding optimal control problem in eq (13) associated with the optimal control variables (u_1^*, u_2^*). Then, there exists adjoint variables, λ_i , for i = 1, . . . , 11,*

satisfying

$$
\begin{aligned}
\frac{d\lambda_1}{dt} =& (\lambda_1-\lambda_2)\epsilon u_1 + (\lambda_1-\lambda_3)\,\beta\, N_S^* f^* + \lambda_1\mu_H,\\
\frac{d\lambda_2}{dt} =& \,(\lambda_2-\lambda_3)\,(1-\theta)\,\beta\, N_S^* f^* + \lambda_2\mu_H,\\
\frac{d\lambda_3}{dt} =& (\lambda_3-\lambda_4 k-\lambda_5\,(1-k))\tau u_2 + (\lambda_3-\lambda_{11})\,\delta_1 + \lambda_3\mu_H - C_1,\\
\frac{d\lambda_4}{dt} =& \,(\lambda_4-\lambda_6)\,\alpha_1 + (\lambda_4-\lambda_9)\,\gamma_1 + \lambda_4\mu_H,\\
\frac{d\lambda_5}{dt} =& \,(\lambda_5-\lambda_7)\,\alpha_2 + (\lambda_5-\rho_1\lambda_8-\lambda_9\,(1-\rho_1))\,\sigma_1 + (\lambda_5-\lambda_{11})\,(1-u_2)\,\delta_2 + \lambda_5\mu_H,\\
\frac{d\lambda_6}{dt} =& \,(\lambda_6-\lambda_9)\,\gamma_2 + \lambda_6\mu_H,\\
\frac{\lambda_7}{dt} =& \,(\lambda_7-\lambda_8\rho_2-\lambda_9\,(1-\rho_2))\,\sigma_2 + (\lambda_7-\lambda_{11})\,(1-u_2)\,\delta_2 + \lambda_7\mu_H,\\
\frac{d\lambda_8}{dt} =& (\lambda_8-\lambda_2)\phi_1 + \lambda_8\mu_H,\\
\frac{d\lambda_9}{dt} =& (\lambda_9-\lambda_2)\phi_2 + \lambda_9\mu_H,\\
\frac{d\lambda_{10}}{dt} =& \,((\lambda_1-\lambda_3)\,S_U^* + (\lambda_2-\lambda_3)\,((1-\theta)\,S_E^*))\,\beta f^* - ((\lambda_1+\lambda_3)\,S_U^* + (\lambda_2+\lambda_3)\,((1-\theta)\,S_E^*))\,\beta \text{[illegible]}\\
& -\lambda_{10}\left(\frac{K_S\Lambda_S - 2\,\Lambda_S N_S^*}{K_S} - \mu_S\right),\\
\frac{d\lambda_{11}}{dt} =& -C_2.
\end{aligned}
\tag{17}
$$

The terminal conditions are

$$\lambda_i(T) = 0, (i = 1, ..., 11). \tag{18}$$

Furthermore, the optimal controls (u_1^*, u_2^*) *satisfy the optimality conditions*

$$
\begin{aligned}
u_1^* =& \max\left(\min\left[\frac{(\lambda_1-\lambda_2)\,\epsilon S_U^*}{C_1}, 1\right], 0\right),\\
u_2^* =& \max\left(\min\left[\frac{(\lambda_3-\lambda_4 k-\lambda_5(1-k))\tau I + ((\lambda_{11}-\lambda_5)T_L^* + (\lambda_{11}-\lambda_7)V_L^*)\delta_2}{C_2}, 1\right], 0\right).
\end{aligned}
\tag{19}
$$

Proof. The adjoint system is obtained using Pontryagin's Principle in [2] as follows:

$$\frac{d\lambda_1}{dt} = -\frac{\partial H}{\partial S_U}, \quad \frac{d\lambda_2}{dt} = -\frac{\partial H}{\partial S_E}, \quad \frac{d\lambda_3}{dt} = -\frac{\partial H}{\partial I}, \quad \ldots \quad , \frac{d\lambda_{11}}{dt} = -\frac{\partial H}{\partial D} \tag{20}$$

and the zero final time conditions (transversality), $\lambda_i(t_f) = 0, i = 1, 2, 3, \ldots, 11$.

Finally, the characterization of the optimal control given by eq (19), is obtain by solving the equations

$$\frac{\partial H}{\partial u_i} = 0, \quad i = 1, 2. \tag{21}$$

Applying the bounds on the controls, we get the preferred characterization. □

The optimality system is made up of the state system eq (13) with the initial conditions, the adjoint system with the transversality conditions and the control characterization.

REFERENCES

1. Fleming WH, Rishel RW. Deterministic and Stochastic Optimal Control. Springer, New York.1975.

2. Pontryagin LS, Boltyanskii VG, Gamkrelidze RV, Mishchenko EF. The Mathematical Theory of Optimal Process.1986; (4). Gordon and Breach, New York.

3. Blayneh K, Cao Y, Kwon H. Optimal Control of Vector-Borne Diseases: Treatment and Prevention, Discrete and Continuous Dynamical Systems Series B.2009; 11(3).

4. Moualeu DP, Weiser M, Ehrig R, Deuflhard P. Optimal control for a tuberculosis model with undetected cases in Cameroon, ZIB-Report.(November 2013); 13-73.
