# Supplementary material for: Control of snakebite envenoming: A mathematical modeling study
Source: PLoS Negl Trop Dis. 2021 Aug 27;15(8):e0009711. doi: 10.1371/journal.pntd.0009711 (PMC8428672; doi:10.1371/journal.pntd.0009711)
Supplement: S1 Table — (PDF) [file pntd.0009711.s003.pdf]

S1 Table Monthly reported data on SBE due to saw scaled viper collected from treatment and research Hospital Kaltungo, Gombe State, Nigeria (from January, 2019 - September, 2020).

**Month**	**I**	**CI**	T_E	CT_E	T_L	CT_L	V_E	CV_E	V_L	CV_L	R_D	CR_D	R_W	CR_W	D	CD
January, 2019	99	99	76	76	7	7	8	8	0	0	4	4	93	93	2	2
February, 2019	118	217	82	158	10	17	4	1	1	1	4	8	114	207	0	2
March, 2019	228	445	185	343	23	40	13	25	0	1	20	28	202	409	6	8
April, 2019	266	711	197	540	39	79	17	42	13	14	25	53	238	647	3	11
May, 2019	206	917	185	725	6	85	6	48	0	14	9	62	195	842	2	13
June, 2019	240	1157	180	905	55	140	19	67	5	19	39	101	194	1038	7	20
July, 2019	260	1417	130	1035	106	246	7	74	9	28	47	148	210	1246	3	23
August, 2019	265	1682	164	1199	67	313	13	87	7	35	21	169	234	1480	10	33
September, 2019	273	1955	198	1397	17	330	11	98	1	36	2	171	265	1745	6	39
October, 2019	263	2218	185	1582	45	375	60	158	27	63	44	215	206	1951	13	52
November, 2019	267	2485	212	1794	44	419	17	175	3	66	5	220	251	2202	11	63
December, 2019	118	2603	84	1878	21	440	22	197	0	66	4	224	111	2313	3	66
January, 2020	81	2684	71	1949	0	440	2	199	2	68	5	229	73	2386	3	69
February, 2020	54	2738	39	1988	10	450	6	205	0	68	3	232	50	2436	1	70
March, 2020	82	2820	63	2051	14	464	65	270	12	80	9	241	68	2504	5	75
April, 2020	192	3012	124	2175	33	497	12	282	6	86	15	256	173	2677	4	79
May, 2020	120	3132	83	2258	6	503	6	288	0	86	1	257	113	2790	6	85
June, 2020	142	3274	117	2375	10	513	64	352	9	95	10	267	124	2914	8	93
July, 2020	253	3527	191	2566	38	551	39	391	0	95	19	286	225	3139	9	102
August, 2020	231	3758	208	2774	10	561	5	396	1	96	10	296	215	3354	6	108
September, 2020	250	4008	198	2972	19	580	15	411	6	102	5	301	236	3590	9	117

[1]

[1] I and CI represent number of SBE and its cumulative; T_E and CT_E represent number of early treatment and its cumulative; T_L and CT_L represent number of late treatment and its cumulative; V_E and CV_E represent number of EAR during early treatment and its cumulative; V_L and CV_L represent number of EAR during late treatment and its cumulative; R_D and CR_D represent number of recovered with disability and its cumulative; R_W and CR_W represent number of recovered without disability and its cumulative; D and CD represent number of death due to SBE and its cumulative.
