# Supplementary figures and images for: Control of snakebite envenoming: A mathematical modeling study
Source: PLoS Negl Trop Dis. 2021 Aug 27;15(8):e0009711. doi: 10.1371/journal.pntd.0009711 (PMC8428672; doi:10.1371/journal.pntd.0009711)

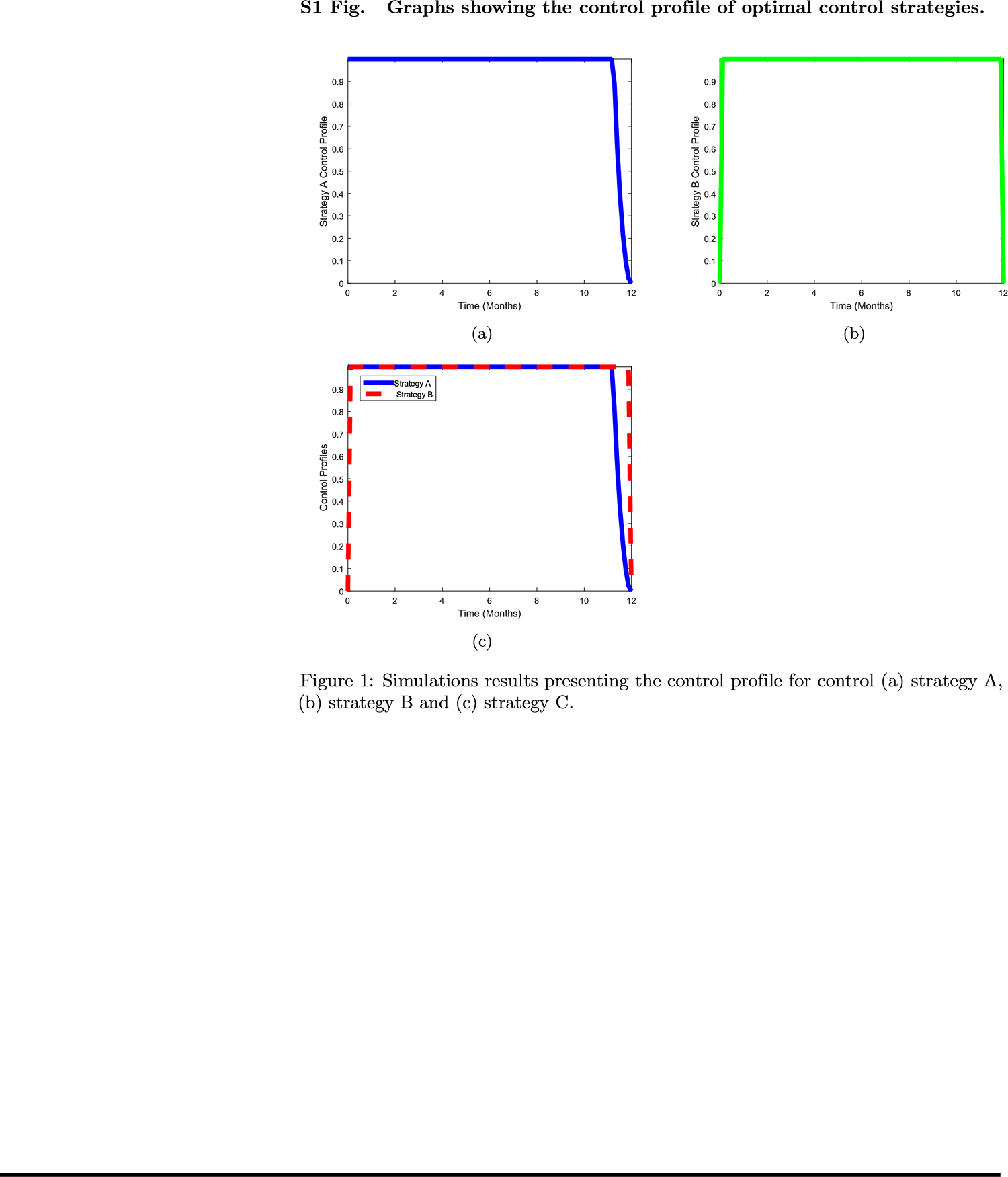

Supplement: S1 Fig — (TIF) [file pntd.0009711.s004.tif]
