# Supplementary material for: Control of snakebite envenoming: A mathematical modeling study
Source: PLoS Negl Trop Dis. 2021 Aug 27;15(8):e0009711. doi: 10.1371/journal.pntd.0009711 (PMC8428672; doi:10.1371/journal.pntd.0009711)
Supplement: Attachment — Submitted filename: Response_Reviwers_08_03_21.docx [file pntd.0009711.s005.docx]

Dear Editor-in-Chief,

We have addressed all the reviewers' comments and revised the manuscript extensively. We are very thankful to the two anonymous reviewers (and the Handling Editor) for their very constructive comments, which have, undoubtedly, significantly enhanced the quality and clarity of the manuscript.

We are really very grateful for this. Below is a detailed description of our response to the comments of the reviewers.

Thank you.

Sincerely,

Nafiu Hussaini

Comments of Reviewers and Responses

**Reviewer #1:** The objectives of the study are clearly articulated. The study design is generally appropriate, with some concerns indicated below. The population selected for this model analysis is adequate, and the statistical mathematical analysis seems appropriate as well. As this is a mathematical modeling study, there are no ethical concerns.

The authors should consider the following aspects in their methodology:

-The terms ‘Enlighted’ or ‘enlightening’ are not the most appropriate to describe the effect of public education campaigns in snakebite envenoming. The authors may consider using alternative terms such as ‘awareness’ or ‘instructed’. The point here is to underscore the effect of these campaigns in the general awareness or information at the community level. In my view ‘enlightening’ has a different meaning. This is presented to the authors for their consideration.

Response:

Thanks. We agree with the reviewer, instead of using susceptible enlightened and unenlightened we used susceptible aware and unware. Also, the term ‘awareness’ has replaced ‘enlightening’ where appropriate.

-Model formulation: When defining the human population, nine categories are proposed and added in the formula. However, the same person may be included in two or more different categories, for example somebody that was ‘enlightened’ and at the same time receive early antivenom treatment. Thus, addition of these categories may provide a mistaken number depending on whether one person may be in two or more categories.

Response:
Thank you for the observation. One of the assumptions of this model is that all the classes are mutual exclusive meaning that no person will belong to two or more different classes at same time. Also, note that the public health enlightenment campaign is targeted at the susceptible individuals. As for the example you presented, if enlightened susceptible person is envenomed then he is no more a susceptible at that time but rather will transit to envenomed class. Further, in the envenomed class there are some possibilities, either he receives early or late treatment with antivenom or does not receive antivenom treatment at all. Therefore, the public enlightenment received might help in seeking for early treatment but it does not imply being in the same category. However, this model can be extended in future to consider treatment seeking behavior of envenomed persons. Also, for better clarification we have added the following statement in the model formulation between lines 62 and 63 and it reads:

“The human population at time t, denoted by N_H_(t), is divided into nine mutual exclusive compartments”

-Method: Control efforts for educating susceptible individuals: the parameter here is whether an individual or groups of individuals were ‘enlightened’ by providing information. But the doubt remains as how to know whether the persons that received the information indeed learned the concepts transmitted and is now ‘enlightened’. It seems that some sort of evaluation of the knowledge acquired should be introduced in the analysis as to provide a follow up of the effective assimilation of the information provided, i.e., a post-hoc evaluation.

Response:

Thanks, in the model formulation we assumed that the parameter θ which can assume values between 0 and 1 both inclusive (i.e.$0\leq\theta\leq1$) measures the efficacy of the public health awareness in reducing snakebite envenoming in the community. If θ = 0, the public health awareness campaign has no effect on the behavior of susceptible individuals, while if θ = 1 then the public health awareness campaign is 100% effective. This information is presented in the description of the model in the supporting document but for clarification we have added this statement to the model assumption (see lines 82 to 87) and it reads as follows:

”It is assumed that the parameter $\theta\in[0,1]$ measures the effectiveness of the public health awareness in reducing snakebite envenoming in the community. If θ = 0, the public health awareness campaign has no effect on the behavior of susceptible individuals, while if θ = 1 then the public health awareness campaign is 100% effective in improving the behavior of susceptible individuals towards taking protective measures against snakebite.”

Fig 2: To help the readers locate the region of study in the map of Nigeria, it is suggested that this map includes also the whole map of Nigeria, highlighting the study area.
Response:

Thanks, we agreed with the reviewer and it has been done as suggested.

In the description of the ‘enlightening’ campaigns, only two modalities are considered, i.e., broadcasting material and mobile technology. However, owing to the variety of cultural settings in Nigeria and similar countries it seems that there should be more options for ‘enlightening’ campaigns, such as **presential** activities in communities, organization of focus groups in schools and other modalities that would adapt to the local contexts. This will increase the cost of these campaigns but will ensure a better communication landscape. The authors may want to include these issues in the discussion of the design and the analysis of the model proposed.

Response:

Thank you. The suggestion is a good one because it might enhance the outcome of the public health enlightenment campaign but the security challenges affecting the study area (North-east Nigeria) hindered such community based campaign in some of the areas. Furthermore, broadcasting media using local languages and mobile technology have yielded positive results with zero security challenges for similar campaign against HIV/AIDS in the same region. For instance campaign for the use of preventive measures such as condom against HIV/AIDS.

The model of Bravo et al. considers the density of the snake population. This variable is not used in the currently proposed model for Nigeria. The authors may want to comment on this, especially since there might be regions in their analysis with a different density of snake populations.

Response:

Thank you very much. The variable representing the total number of venomous snake at time t in the study area is denoted by N_S_(t) and included in the model equations. However, due to the security challenges in some of the places in the study area, we did not go to the field to count the number of snake in the area as in the case of Bravo *et al*. Thus, the number of snake responsible for majority of snakebites in the study area were estimated by fitting the model to real data collected. This approach is also allowed and adequate in mathematical modeling.

**Reviewer #2:** -Are the objectives of the study clearly articulated with a clear testable hypothesis stated?

The study main objective is to apply epidemiological models to then perform optimal control to reduce burden of snakebite mortality and incidence taking into account the cost of performing prevention strategies and improving antivenom delivery.

-Is the study design appropriate to address the stated objectives?

Authors used an adequate epidemiological model, but the box diagram must be improved. It looks tight and is difficult to read. Then, authors adjusted the model by using parameters from literature and by fitting other parameters with MCMC, which is an adequate methodology. They MUST explain why they assumed the saw scaled viper as the only venomous snakes in their total venomous snakes’ population. Finally, their optimal control strategy is clear, but they MUST explain deeply the objective function, and why they summed weighted incidence and mortality with their strategies (These variables doesn't have the same dimensions, so the weight variables B1, B2 and Ci must be explained deeplier).

Response:

Thank you. The schematic diagram has been improved in terms of size and readability as suggested.

Numerous researches have established that the saw scaled viper accounted for the majority of snakebites cases in the study area. For instance, according to the work by Yusuf et al., (2015), saw scaled viper accounted for 95% of the snakebite cases in Norths-Eastern Nigeria (A Hospital Based Survey). Thus, we collected data related to saw scaled viper only. Furthermore, we need to be specific in order to calculate the life expectancy for the snake.

Note that the objective functional depends on one or more of the state and the control variables. Also the objective functional is specified by an integral of the state and/or control variables. Therefore, we have followed the standard way of presenting and defining the objective functional.

The objective functional is given by equ(5), the constraint is also given in equ(4). Further all the components of the objective functional were explained in details.

B_1_ and B_2_ are not variables but constants. These are positive balancing coefficients (weights) that are used to normalize the optimal control. While the weight coefficients, C_1_ and C_2_, are measures of the relative costs of the interventions associated to the control u_1_ and u_2_, respectively. However, we added the following statements between lines 112 and 118 to clarify the objective functional.

“To explore the optimal level of efforts that would be required to control snakebite envenoming in the study area, we constructed an objective functional J(u_1_, u_2_), whose goal is to minimize the number of snakebite envenoming individuals at time t, given by I(t), the cumulative number of snakebite induced death at time t, denoted by D(t) and the costs of applying the control efforts, u_1_ and u_2_, on public health education campaign for susceptible individuals and treatment of envenomed victims, respectively.”

-Is the population clearly described and appropriate for the hypothesis being tested?

They used as a case study northeast Nigeria, a place that has a high burden of snakebite, so the study area and population is appropriate.

-Is the sample size sufficient to ensure adequate power to address the hypothesis being tested?

The sample size is not explicit, because it is not clear if they worked with disaggregated data from the six states of northeast Nigeria, or if they aggregated data. This fact must be explained deeplier.
Response:

Yes, the data are aggregated from the all the six states in the study area. Note that the total number of snakebite cases for twenty-one months were collected from the regional snakebite treatment and research center at Kaltungo located in the study area. Then the cumulative number of cases over these periods were used in fitting the model and performed other numerical simulations. It is important to note there are six states in the study area and that all the cases of snakebite across these states were obtained from the treatment center. This was done by identifying the state of origin of all the victims who attended the regional snakebite treatment and research center. We have added the following statements between lines 156 and 160 under data collection.

“The cumulative number of these seven different sets of data on snakebite cases will be used to fit the model as well as to performed some numerical simulations. Thus, the data used in this work are aggregated from all the six states that made up the study area. Note that all the seven sets of snakebite data collected are due to saw scaled viper (carpet viper).”

-Were correct statistical analysis used to support conclusions?

MCMC confidence intervals seems adequate, but authors do not talk about the convergence of the chains of the algorithm. May be using a gelman-rubin diagnostic. Also, the optimal control strategy doesn't have the evidence of the convergence.

Response:

Thanks, we have used the Gelman-Rubin diagnostic test to show the convergence of the chains as suggested. The outcome of Gelman-Rubin diagnostic test is presented in Table 5 and the following statements between lines 174 and 182 were incorporated under model fitting.

“A set of results is estimated using the least square method with 100,000 number of iterations and the outcome is employed as initial guess for the MCMC method. To ensure the convergence of MCMC algorithm we used Gelman-Rubin diagnostic test implemented in MATLAB. We set the number of iteration to be 80000 with a burn-in of 40000 iterations. According to Gelman and Rubin [59], if chains have converged to the target posterior distribution, then Potential Scale Reduction Factor (PSRF) denoted by (Rc) should be sufficiently close to 1. The result in Table 5 shows that the (Rc) values are between 0.99 to 1.04 and thus all the chains have converged.”

For the convergence optimal control, we have added a subsection (see lines 232 to 256) describing the procedure for solving an optimality system and elaborated more on the numerical simulations. See as follows:

Procedure for Solving Optimality System

In order to obtain solution for the optimality system which consists state equations eq(4), adjoint system eq(23), the characterizations eq(25) and corresponding initial/final conditions, we apply the Runge-Kutta fourth order technique which is more accurate. It is a multiple-step method and also known as forward-backward sweep method (for detail description of this technique see [60]). The procedure starts with an initial guess on the control variable given initial conditions for the state variables, the solutions for the state equations will be approximated using the Runge-Kutta forward sweep technique. Given the state solutions from the preceding step and the final time conditions for adjoints, the solutions for adjoint equations will then be approximated using Runge-Kutta backward sweep method. The value of control variables is updated by taking the average of the preceding value and the new value arising from the control characterization. The procedure is repeated for forward numerical scheme and updating the controls until successive values of all states, adjoints, and controls converge.

Numerical Simulation of the Optimal Control Model

The numerical solutions of the consequential optimality system obtained in S2 File are carried out. The forward-backward sweep method is employed using the initials conditions and parameter values in Tables 3 and 4, with ($\sigma_{1}=\sigma_{2}=0.45,;\theta=\varepsilon=0.65;k=0.55$). The algorithm starts with an initial guess of (u_1_, u_2_) = (0, 0) for the optimal controls and the state variables are then solved forward in time using Runge-Kutta method of the fourth order. Further, the state variables and initial control guess are used to solve the adjoint equations eq(23) backward in time with the given final condition eq(24), using the backward fourth order Runge-Kutta method. The controls u_1_ and u_2_ are then updated and used to solve the state equations and then the adjoint system. This iterative process ends when the solutions converge.

-Are there concerns about ethical or regulatory requirements being met?

No.

--------------------

<b>Results</b></br></br>
-Does the analysis presented match the analysis plan?</br>
-Are the results clearly and completely presented?</br>
-Are the figures (Tables, Images) of sufficient quality for clarity?</br></br>

**Reviewer #1:** The analysis presents match well with the analysis plan. The results are clearly and completely presented. The figures and tables could be improved along the lines indicated below.

Effects of public health enlightenment campaigns: In addition to the objective criteria of reduction in SBE as an outcome of the enlightening campaigns, it would be interesting to consider evaluation of improvement in knowledge on how to prevent snakebites through instruments that follow up the knowledge acquired by people that attended or benefited from the campaigns.

Response:

Agreed, but it will require another set of data to be collected. So we make that to be part of our future study.

The variables ‘enlightening’ and early treatment of snakebites are considered as separate parameters in the analysis. However, there seems to be a clear link between them, since it is likely that people that benefit from the enlightening campaigns would also be people that would procure an early attention to snakebites, assuming that they will not look for traditional treatments that delay the access to health facilities.

Response:
Agreed, there might be link between them but they are different state variables in the proposed model. As we said earlier the model can be extended in future by incorporating treatment seeking behaviors of envenomed persons.

Table 14: The meaning of the heads of each column should be indicated in a foot note, i.e., the meaning of I, CI, TE, CTE, etc.

Response:

This is done and is presented as follows:

• I and CI represent number of SBE and its cumulative.

• TE and CTE represent number of early treatment and its cumulative.

• TL and CTL represent number of late treatment and its cumulative.

• VE and CVE represent number of EAR during early treatment and its cumulative.

• VL and CVL represent number of EAR during late treatment and its cumulative.

• RD and CRD represent number of recovered with disability and its cumulative.

• RW and CRW represent number of recovered without disability and its cumulative.

• D and CD represent number of death due to SBE and its cumulative.

**Reviewer #2:** -Does the analysis presented match the analysis plan?

Results are great, graphics could be improved in aesthetics, but their contests match the analysis plan. Just as said before, authors must go deeplier into the convergence of MCMC chains and their optimal control strategy.

Responded already.

-Are the results clearly and completely presented?

The paper has a strongly mathematical-biased language, which must be changed to be more comprehensible for the multi-disciplinary audience of PLOS NTDS. Even so, results and conclusions fits totally the requirements of originality, importance and rigorous methodology required by the journal.

-Are the figures (Tables, Images) of sufficient quality for clarity?

Images have a high-quality methodology and importance behind them, but they are not clearly explained and their aesthetics can be improved. The box-diagram of the epidemiological model seems of bad quality, and it looks tight. The figures 3 to 5 must be explained better in the image caption: What do you want to point with these plots? Do the model fit well de data? What is the impact of the level of education? What means these strategies A and B in terms of the results shown in Figure 5, what strategy is better? Are necessary the control profiles, or they can go to supplementary materials? These profiles must be explained deeplier in the caption of the figure.

Response:

We have explained the figures in the image caption and the figures showing the control profiles for the optimal control strategies have been moved to supplementary document as suggested. See the modified caption for Figures 3 to 5, as follows:
Figure 3: Fitting of the snakebite model with the reported cumulative number of (a)snakebite envenoming (b) early treatment (c) late treatment (d) EAR during early treatment (e) EAR during late treatment (f) recovered with disability (g) recovered without disability and (h) snakebite deaths.

It can be seen that the model fitted well with the respective data sets collected from the snakebite treatment and research center.

Figure 4: Simulations showing the impact and effectiveness of public health awareness in averting number of (a) snakebite envenoming (b) snakebite induced death (c) disability and (d) simulations showing the impact of early treatment with antivenom on snakebite induced death. In Figures (a), (b) and (c) the red, black, blue and green colors correspond to 0%, 10%, 50% and 90% public health awareness campaign coverage and its efficacy respectively. While in Figure (d), the red, black, blue and green colors represent 0%, 10%, 50% and 90% of envenoming individuals receiving early treatment with antivenom.

Figure 5: Simulations results of optimal control model showing the effectiveness of

(a) strategy A in averting the number of snakebite induced deaths as against without strategy

(b) strategy B in averting the number of snakebite induced deaths as against without strategy

(c) strategy C in averting the number of snakebite induced deaths as against without strategy,

(d) strategy A in averting number of snakebite envenoming as against without strategy. In the graphs the solid line denotes without control strategy while dotted line indicates with control strategy.
--------------------

<b>Conclusions</b></br></br>
-Are the conclusions supported by the data presented?</br>
-Are the limitations of analysis clearly described?</br>
-Do the authors discuss how these data can be helpful to advance our understanding of the topic under study?</br>
-Is public health relevance addressed?</br></br>

**Reviewer #1:** The conclusions are generally supported by the data and analyses done, although there are some issues indicated above that need to be considered.

The authors may consider to include, at the end of their manuscript, a section discussing the limitations of the analysis.

Response:

Thanks, the section for limitation of the study has been added between lines 385 and 388 and reads as follows:

Limitation of the Study

This study has the following limitations. Owing to the security challenges in some of the places in the study area, the total population of saw scale viper was estimated not counted. The seasonality and temporal dynamics of the envenoming are not considered. The data did not capture unreported cases in the study area.

May The method proposed and described has evident implications from the public health perspective, as it provides interesting tools to model issues related to snakebite envenoming and ways to reduce their impact, including cost-effectiveness analysis.

**Reviewer #2:** -Are the conclusions supported by the data presented?

Yes, but they could be more direct: ii) What is that substantial number of deaths reduced by that strategy? iii) How many DALYs and deaths will be reduced by combining strategies?

Response:

Thanks, this has been addressed as follows:

ii) If at least 50% of SBE patients received early treatment more than 900 cases of death will be averted in the study area.

iii) The implementation of the control strategies either separately or in combination will help in reducing the number of death in the community. However, the combination of the two control strategies averts more than 7,227 deaths and 169,205 DALYs in the population.

-Are the limitations of analysis clearly described?

No. They don't state the limitations of only using population dynamics of one venomous snake, or the possible effect of seasonality and temporal dynamics of the envenoming, or the limitations of the assumptions behind the proposed model, or what happen with underreporting. A limitations section must be added.

Response:

This has been addressed.

-Do the authors discuss how these data can be helpful to advance our understanding of the topic under study?
-Is public health relevance addressed?

Not so much. This work has a GREAT potential, but that potential is not stated directly. Maybe at the end of conclusions you should add that is the first time that a study mixing epidemiological modeling with optimal control is applied to snakebite, and how can this framework be extrapolated to other countries, and how to use it with fragmented or incomplete datasets.

Response:

Thanks, the suggestion has been included at the end of conclusion between lines 380 and 383 and it read as follows:

To the best of the authors' knowledge, this is the first time a study mixing epidemiological modeling with optimal control is applied to snakebite. The model can also be used to assess snakebite envenoming in other settings or countries of high incidence.
