# Supplementary material for: Control of snakebite envenoming: A mathematical modeling study
Source: PLoS Negl Trop Dis. 2021 Aug 27;15(8):e0009711. doi: 10.1371/journal.pntd.0009711 (PMC8428672; doi:10.1371/journal.pntd.0009711)
Supplement: Attachment — Submitted filename: Response to Reviewers_Comments.docx [file pntd.0009711.s006.docx]

The table of the parameters in the methodology does not have units. Please add them similar to the table of the estimated parameters in results.

Response:

It has been addressed as suggested. See Table 2.

Why does u_1_ and u_2_ are squared in the objective function if both variables are between 0 and 1? It is not totally clear why do you want to have a cost function non-linear.

Response:

Quadratic cost function is mostly used in optimal control problem in epidemiology, because quadratic cost function is convex with respect to the control variables. Thus, it helps in fulfilling the convexity requirement of the integrand of the objective functional. Furthermore, Colaneri et al., (2014) stated that optimality conditions of an optimal control problem can be guaranteed if the cost functional is convex with respect to the control variables. Also, the used of quadratic cost on the controls is in line with numerous studies in the literature (see Joshi, 2002; Adams et al., 2004; Okosun et al., 2013; Agusto et al., 2014; Rodrigues et al., 2014). However, for clarity we have added the following statements between lines 120 and 124, and it reads

“**In line with Rodrigues et al., [62], Agusto et al., [63] and the references therein, we used a quadratic cost functional with respect to the control variables *u*_1_ and *u*_2_ in order to guarantee convexity condition for optimality mentioned in Colaneri et al., [61]**.”

Why does the factor of u_1_ and u_2_ in the objective function have no weight? (Or weight =1?) Please explain this.

Response:

u_1_ and u_2_ are the control variables. In the objective functional given by, the quantities C_1_ and C_2_ are the relative costs weight constants associated with the controls u_1_ and u_2_, respectively. Therefore, the controls have weight constants and is not 1 (see lines 129 -136). In the numerical simulations we used C_1_=US$0.28 i.e. per capita cost on public health awareness and C_2_=US$237 i.e. per capita cost for treatment with antivenom therapy (see Table 4).

The block diagram quality and map of study area is low; it pixelates when zoom is applied. Please upload a higher resolution version.

Response:

This is done as suggested.

Reviewer #2: Why is the cost function of Cost-effectiveness analysis different than the one presented in the methodology (Objective function?) This function does not have the squares that are present in the method cost function. Please explain deeplier this new function and its differences with cost function of the methodology.

Response:

For the purpose of carrying out cost effectiveness analysis of the control strategies, we decided to use a linear cost function with respect to controls variables u_1_ and u_2_ which is more practical and for the benefit of computational convenience. It is important to note that in the computation of cost effectiveness of the control strategies the cost function with respect to controls is not required to be convex. Also the cost functions constructed is in line with the cost functions in Elbasha et al., (2008), Hove-Musekwa et al., (2014), Adamu et al., (2020). Another difference is the discount term e^-rt^ which is included for discounting the value of the future cost accruing over the given finite planning horizon. However, for the purpose of clarification we have added the following statement between lines 304-306, and is stated as follows:

**“In line with Hove-Musekwa et al., [23] and Adamu et al., [54], a linear cost function with respect to the control variables *u*_1_ and *u*_2_ of the cost effectiveness analysis for the control strategies is used.”**

Please explain deeplier how did you computed the DALYS.

Response:

Following Habib, et al., [38] and Hamza et al., [40], 23.41 discounted DALYs approximation is equivalent to one early mortality due to snakebite. Therefore, the deaths averted by each strategy shown in Table 9, column 2, were converted to DALYs by taking the product of the total number of deaths averted by each Strategy and 23.41 DALYs and the results are shown in Table 11 column 2. For example, implementing strategy A only averts 5,857 deaths see Table 9. Thus, the total DALYS averted by strategy A = 23.41 X 5857 = 137,112.37 see Table 11 column 2. However, for the purpose of clarity, we have added the following statements between lines 334 – 339 and reads as follows:

“**Following Habib, et al., [38] and Hamza et al., [40], 23.41 discounted DALYs approximation is equivalent to one early mortality due to snakebite. Therefore, the deaths averted by each strategy shown in Table 9, column 2, were converted to DALYs by taking the product of the total number of deaths averted by each Strategy and 23.41 DALYs and the results are shown in Table 11 column 2.**”

Reviewer #2: Please state more limitations of the model, as which limitations must be taken into account for its application in other countries? In terms of the assumptions, the species, the available data needed to calibrate the model and perform optimal control, and how can be these limitations overcome.

**Response:**

**We have added the following under the limitation between lines 401- 411 and it reads:**

**“This study has the following limitations. Our model could be extended to include some other important epidemiological and demographic features like the spatial and temporal dimensions of human-snakes interaction and also the impact of seasonality on the dynamics of envenoming. Furthermore, the data we collected did not capture unreported cases in the study area and the total population of saw scale viper was estimated not counted. The assumption that only saw scale viper is considered could be relaxed to include multiple species of snakes provided the relevant data could be obtained. Also more control variables could be incorporated into the model to take care of other possible control measures like use of snake repellent, etc.**”
